# Meeting

**Published:** 1877-03

**Authors:** 


### MEETING.

The Mississippi Valley Dental Society has just closed one of the most interesting and profitable sessions ever held by that body.

A programme of important practical subjects was presented for consideration and discusssion, in which a large number took part.

There was a larger number present than for several years past. There was an increase in membership.

The proceedings will be published in a future number of the REGISTER.

